# Support for a ban on characterizing flavours in cigarettes in the UK: a longitudinal online survey

**DOI:** 10.1093/eurpub/ckae181

**Published:** 2024-11-19

**Authors:** Crawford Moodie, Catherine Best, Daniel Jones

**Affiliations:** Institute for Social Marketing and Health, Faculty of Health Sciences and Sport, University of Stirling, Stirlingshire, Grange Road, United Kingdom; Institute for Social Marketing and Health, Faculty of Health Sciences and Sport, University of Stirling, Stirlingshire, Grange Road, United Kingdom; Institute for Social Marketing and Health, Faculty of Health Sciences and Sport, University of Stirling, Stirlingshire, Grange Road, United Kingdom

## Abstract

Most European countries have banned flavoured cigarettes. No study has explored whether support for such a ban changes post-implementation. In the UK, a menthol cigarette ban came into force in May 2020. A longitudinal survey in the UK with adult smokers and ex-smokers explored support for the ban in 2019 (*N* = 3175) and 2022 (*N* = 3047). Support increased from 2019 (pre-ban) to 2022 (post-ban) for all participants (18.1% to 35.0%), with increased support evident for flavour cigarette smokers (20.7% to 34.5%), non-flavour cigarette smokers (13.5% to 18.1%), and ex-smokers (24.4% to 50.8%). Increased support for tobacco-related policies helps regulators defend policy decisions.

## Introduction

More than 30 countries have banned flavours or characterizing flavours in cigarettes [1]. This is primarily due to the Tobacco Products Directive (TPD), which banned characterizing flavours in cigarettes (including hand-rolled) across the European Union [2]. The ban on characterizing flavours in cigarettes came into force in May 2016, with menthol cigarettes, as a consequence of having more than 3% of the tobacco market, granted an extension until May 2020 under Article 7 of the TPD [2]. The ban on characterizing flavours, including menthol cigarettes, was incorporated into the UK law prior to Brexit.

A systematic review and meta-analysis found only two pre-post studies in Europe to have explored consumer response to the ban on menthol cigarettes in 2020 [3]. The first, longitudinal research in the Netherlands with adult smokers and ex-smokers, found a decrease in use of menthol cigarettes following the ban, and an increase in quit attempts and quitting among pre-ban menthol smokers [4]. The second, a repeat cross-sectional study with 16–19-year-olds in England, found menthol or capsule cigarette use decreased from February 2020 (pre-ban) to August 2020 (post-ban) [5].

With respect to support for a ban on flavours in cigarettes, cross-sectional data with adult smokers from eight European countries pre-ban found that approximately half (46.3%) supported a ban on additives, including flavourings, in cigarettes [6]. Several studies, particularly in the USA, have also explored support for a hypothetical ban on the sale of flavoured tobacco products [7]. Few studies have explored post-ban support, e.g. a survey with young adult ever tobacco users in San Francisco (*N* = 247), conducted following a ban on all non-tobacco flavoured tobacco products and e-cigarettes, found that only 8.1% supported the ban [8]. We are unaware of any research in Europe or elsewhere exploring support before and after a ban on flavours in cigarettes. Given public support can help regulators justify the decision to have introduced a policy, we explored whether support for the flavour ban changed in the UK post-implementation.

## Methods

### Design and sample

The ‘Adult Tobacco Policy Survey’ is a longitudinal study with 6233 cigarette smokers in the UK recruited between April and May 2016 (Wave 1) and followed up, irrespective of whether they continued to smoke, in September–November 2017 (Wave 2), May–July 2019 (Wave 3), and October–November 2022 (Wave 4). Participants were drawn from the online panel of YouGov, a market research company. Participants received a small incentive, in the form of points that could be subsequently exchanged for vouchers, for participation at each wave.

We explored support for the flavour ban between Wave (W) 3 and W4. Of those recruited at W1, 3175 responded at W3 (including 2412 cigarette smokers and 700 ex-smokers) and 3047 at W4 (including 1935 cigarette smokers and 1043 ex-smokers). Cigarette smokers were those who reported being a current smoker and smoking cigarettes (either factory-made or hand-rolled) in the last 3 months. The sample at W1 was weighted by age, gender, government office region, and tobacco consumption to represent the national profile of smokers aged 16 and over in the UK, with subsequent wave weights adjusted for sample attrition.

### Procedure

Participants were asked at W3 ‘To what extent, if at all, do you agree or disagree that the sale of flavoured cigarettes and rolling tobacco, including menthol and capsule cigarettes, should be banned*?*’, and at W4 ‘To what extent, if at all, do you agree or disagree with the ban on the sale of flavoured cigarettes and rolling tobacco, including menthol and capsule cigarettes?’. Response options ‘Strongly agree’, ‘Agree’, ‘Neither agree nor disagree’, ‘Disagree’, ‘Strongly disagree’, and ‘Don’t know’ were collapsed into approval (Strongly agree/Agree), neutral (Neither agree nor disagree/Don’t know), and disapproval (Strongly disagree/Disagree).

To assess change over time we examined approval as a binary outcome of strongly agree or agree versus other responses. Flavour cigarette smokers were defined as: cigarette (factory-made or roll-your-own) smokers who indicated that their usual (or current) brand had tobacco and menthol, or tobacco and some other flavour; factory-made cigarette smokers who smoked cigarettes with a capsule in the filter that could be burst to change the flavour; or roll-your-own smokers who used filters that were flavoured or had a capsule that could be burst to change the flavour. Ex-smokers were those that indicated that they had stopped smoking cigarettes completely. Ethical approval was granted by the General University Ethical Panel at the University of Stirling (GUEP 8359).

### Analysis

Change across survey waves was assessed using generalized estimating equations with exchangeable correlation structure and robust standard errors. The analysis, which included the full sample, was adjusted for smoking status.

## Results

Approval increased for all participants, from 18.1% at W3 to 35.0% at W4. Support among non-flavour cigarette smokers at W3 increased from 13.5% at W3 to 18.1% at W4, and for flavour cigarette smokers at W3 from 20.7% at W3 to 34.5% at W4 (see Table 1). Among ex-smokers, approval was 24.4% at W3 and 50.8% at W4. Generalized estimating equations, adjusted for smoking status, showed a significant increase in the proportion approving of the ban across W3 and W4 (odds ratio = 1.52, 95% confidence interval 1.37–1.70).

**Table 1.** Pre- and post-ban support for a ban on characterizing flavours in cigarettes among cigarette smokers and ex-smokers

		Wave 3 (pre-ban)	Wave 4 (post-ban)
Cigarette smokers and ex-smokers	*Approval*		
	*n*	488	698
	Weighted %	18.1%	35.0%
	95% CI	16.0–20.4%	23.3–48.8%
	*Neutral*		
	*n*	1391	1342
	Weighted %	38.9%	33.3%
	95% CI	36.5–41.4%	26.8–40.6%
	*Disapproval*		
	*n*	1296	1007
	Weighted %	43.0%	31.7%
	95% CI	40.4–45.7%	25.2–39.0%
Non-flavour cigarette smokers at W3	*Approval*		
	*n*	210	247
	Weighted %	13.5%	18.1%
	95% CI	10.9–16.6%	15.7–20.7%
	*Neutral*		
	*n*	875	655
	Weighted %	44.7%	50.4%
	95% CI	41.3–48.2%	46.9–53.9%
	*Disapproval*		
	*n*	663	350
	Weighted %	41.8%	31.5%
	95% CI	38.2–45.4%	28.3–35.0%
Flavour cigarette smokers at W3	*Approval*		
	*n*	102	91
	Weighted %	20.7%	34.5%
	95% CI	16.1–26.1%	19.1–54.1%
	*Neutral*		
	*n*	199	140
	Weighted %	28.0%	25.2%
	95% CI	23.0–33.6%	17.9–34.3%
	*Disapproval*		
	*n*	363	224
	Weighted %	51.3%	40.3%
	95% CI	45.5–57.2%	29.0–52.6%
Ex-smokers	*Approval*		
	*n*	176	367
	Weighted %	24.4%	50.8%
	95% CI	20.2–29.2%	31.1–70.3%
	*Neutral*		
	*n*	317	454
	Weighted %	38.5%	25.7%
	95% CI	33.6–43.7%	16.3–38.1%
	*Disapproval*		
	*n*	270	291
	Weighted %	37.1%	23.5%
	95% CI	32.3–42.1%	14.0–36.6%

## Discussion

Among cigarette smokers (whether flavoured or non-flavoured) and ex-smokers, approval for the ban on flavour cigarettes increased post-implementation. The highest increase in support was from ex-smokers. Increased support among ex-smokers has been found following the introduction of other tobacco control policies, such as standardized packaging [9]. As smoking prevalence has declined in the UK, as it has in many other countries, there are now more ex-smokers than smokers in the population. Ensuring that this population does not relapse is key to continuing to lower smoking prevalence. While we did not ask participants why they supported the policy or otherwise, a possible area for future research, it is likely that ex-smokers support policies that they perceive as helping them to prevent relapse.

Increased post-implementation support has also been found for other tobacco-related policies, including pictorial warnings on packaging, standardized packaging, a ban on the open display of tobacco in retailers, and smoke-free public places [10]. Increased support, particularly among smokers, as the population most likely to be resistant to tobacco-related policies, helps governments defend policy decisions and may encourage them to introduce further policies. It may also encourage other governments to introduce similar measures given that public opinion is an important determinant of policy change in democratic countries and one which can facilitate tobacco policies by increasing political will for implementation [9, 10].

While the first longitudinal study to explore support for a flavour ban pre- and post-implementation, the study does not provide any insight into longer-term response. The data are reliant upon self-report. Socially desirable responding may have influenced responses at one or both waves. Those in the most deprived populations have lower levels of regular internet access and may be less likely to be part of online panels. This, and attrition across waves, may have skewed findings. Nevertheless, the findings extend the literature on flavour bans and support for tobacco-related policies.

Conflict of interest: None declared.

## Data Availability

The data that support the findings of this article will be available from the corresponding author, Crawford Moodie, upon reasonable request, from April 2025. Key points No study has explored whether support for a ban on flavour cigarettes changes following its implementation. Approval for the ban on characterizing flavours in cigarettes in the UK increased and disapproval decreased post-implementation among cigarette smokers (whether flavoured or non-flavoured) and ex-smokers. Demonstrating increased support for tobacco-related policies post-implementation helps governments defend the decision to introduce these policies.
